# Implementation of a double-grating interferometer for phase-contrast computed tomography in a conventional system nanotom^®^ m

**DOI:** 10.1063/1.5022184

**Published:** 2018-01-26

**Authors:** Anna Khimchenko, Georg Schulz, Peter Thalmann, Bert Müller

**Affiliations:** Biomaterials Science Center, University of Basel, 4123 Allschwil, Switzerland

## Abstract

Visualizing the internal architecture of large soft tissue specimens within the laboratory environment in a label-free manner is challenging, as the conventional absorption-contrast tomography yields a poor contrast. In this communication, we present the integration of an X-ray double-grating interferometer (XDGI) into an advanced, commercially available micro computed tomography system nanotom^®^ m with a transmission X-ray source and a micrometer-sized focal spot. The performance of the interferometer is demonstrated by comparing the registered three-dimensional images of a human knee joint sample in phase- and conventional absorption-contrast modes. XDGI provides enough contrast (1.094 ± 0.152) to identify the cartilage layer, which is not recognized in the conventional mode (0.287 ± 0.003). Consequently, the two modes are complementary, as the present XDGI set-up only reaches a spatial resolution of (73 ± 6) *μ*m, whereas the true micrometer resolution in the absorption-contrast mode has been proven. By providing complimentary information, XDGI is especially a supportive quantitative method for imaging soft tissues and visualizing weak X-ray absorbing species in the direct neighborhood of stronger absorbing components at the microscopic level.

## I. INTRODUCTION

Micro computed tomography (μCT) based on the conventional X-ray sources usually operates in the absorption-contrast mode. Disadvantages of this technique are a limited contrast in materials composed of low atomic number elements and acquired values cannot be easily related to a local X-ray absorption as determination of an effective photon energy of a polychromatic spectrum is complex. Simultaneous visualization of soft and hard tissues is a challenge too. For an optimized choice of the photon energy, weak absorbing parts do not provide sufficient contrast, and thus, staining procedures are often required,^1,2^ whereas hard parts show the streak and beam hardening artifact characteristics for low photon energies.

In the hard X-ray regime, phase contrast is often preferred over conventional absorption contrast for soft tissue imaging^3,4^ and visualizing weak X-ray absorbing species in the direct neighborhood of stronger absorbing components,^5^ in particular, for three-dimensional imaging of a cartilage which is involved in the degenerative changes of a joint,^6–10^ providing imaging data of morphological features with unprecedented contrast. Disorders associated with cartilage degeneration, such as debilitating joint diseases, are one of the leading causes of disability worldwide.^10,11^ The ability to assess the pathological changes at the cellular level non-destructively, with time-efficiency, and without the use of contrast agents^12^ within a laboratory environment can be beneficial for a broad range of medical and biomedical applications, opening research avenues in the field of diagnostic, tissue engineering, and regenerative medicine.^13–15^

Several research teams have built computed tomography systems working in the phase-contrast mode.^16–19^ Grating interferometry is a phase-contrast imaging technique especially powerful with regard to its quantitativeness and distinctive contrast, even if polychromatic sources are used.^20^ As the pixel sizes of commercially available detectors are generally too large to resolve the interference pattern directly, as required for a single-grating set-up,^21^ grating interferometry is preferentially performed in two- and three-grating configurations. The three-grating set-up, Talbot-Lau interferometer, works with conventional sources.^22–24^ However, introduction of the source grating to enhance a spatial coherence reduces an available photon flux. Measurements in a Talbot configuration with two gratings are performed with micro-focus tubes^25^ and multiline^26^ and liquid-metal-jet^27^ sources. The extension of a commercially available μCT system by a grating set-up is particularly interesting, as phase contrast leads to complementary information, which enables multi-modal imaging in a single advanced apparatus without the requirement of building a complete system. So far, there has been no detailed study on the Talbot interferometer realization within a commercial laboratory absorption-contrast μCT system with a transmission polychromatic source, potentially due to the restrictions in system dimensions, limited flux, and micrometer-sized X-ray sources.

We propose integration of a symmetrical^24^ X-ray double-grating interferometer (XDGI) into an advanced μCT system nanotom^®^ m (GE Sensing & Inspection Technologies GmbH, Wunstorf, Germany). The purpose of the present study is to make a direct comparison between phase- and absorption-contrast tomographies performed within the laboratory μCT system while analyzing a human knee joint sample in order to highlight the added value of the extension by a grating set-up.

## II. RESULTS AND DISCUSSION

**FIG. 1.**
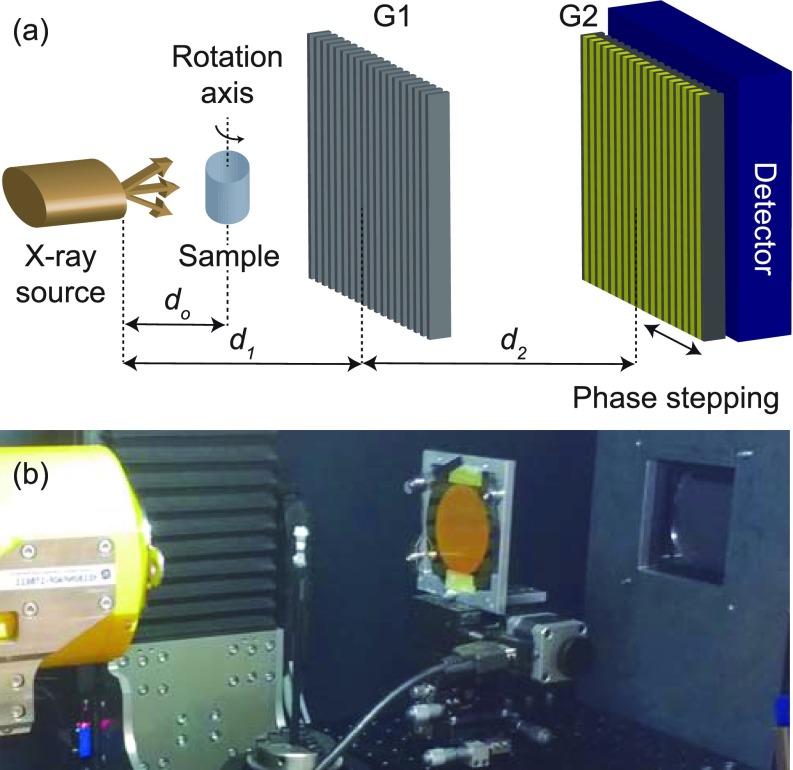
(a) Scheme and (b) realization within the μCT system nanotom^®^ m of a double-grating symmetric (*d*_1_ = *d*_2_) interferometric set-up. G1: phase grating; G2: analyzer grating; *d_o_*: distance from the source to the sample; *d*_1_: distance from the source to phase grating G1; *d*_2_: distance from the phase grating G1 to analyzer grating G2.

A double-grating symmetric interferometric set-up,^28^ see Fig. 1, was incorporated into the advanced μCT system nanotom^®^ m with an adjustable focal spot diameter *w* (0.9–2.7 *μ*m). The main advantages of the symmetrical set-up (*d*_1_ = *d*_2_, where *d*_1_ denotes the distance from the source to the phase grating and *d*_2_ from the phase grating to the analyzer grating) compared to the asymmetrical (*d*_1_ ≠ *d*_2_) one are the straightforward grating positioning, flexibility, and set-up cost reduction as the same mask can be used for fabricating both gratings.

**FIG. 2.**
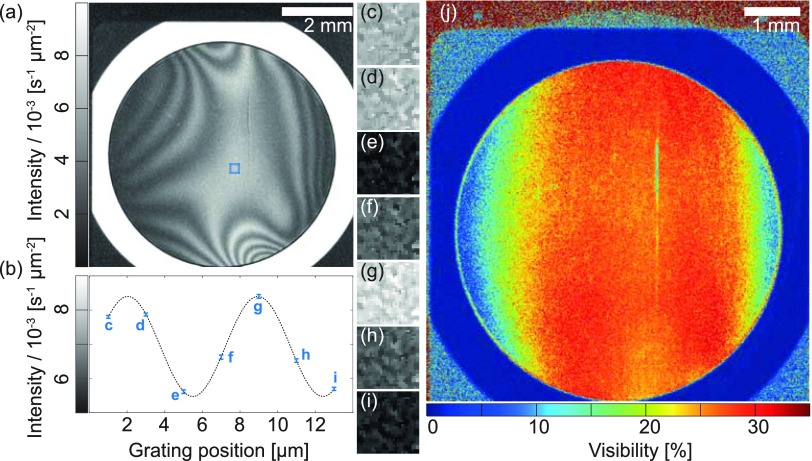
Interference pattern at the first fractional Talbot distance. (b) Stepping curve: Mean intensity oscillations in the center of the field of view (30 × 30 detector pixels) over seven grating positions [(c)–(i)]. (j) The visibility map highlights that for the proposed set-up shadowing effects can be neglected. The scan was performed with a focal spot diameter of *w* = 2.0 *μ*m.

An interference pattern at the first fractional Talbot distance is shown in Fig. 2(a). The stepping curve given in Fig. 2(b) shows the mean intensity oscillations in the center of the field of view (30 × 30 detector pixels) over seven grating positions [Figs. 2(c)–2(i)]. Analyzing the visibility^29^ map, see Fig. 2(j), one can directly observe a relatively large (3 cm × 6 cm) homogeneous region with an average visibility of 25%. Thus, for the proposed set-up with the selected source-to-G2 distance, grating diameter, and design energy, the shadowing effects causing the decrease in visibility towards lateral directions can be neglected and curved gratings^18^ are not required. The mean visibility in the center of the field of view was (25.6 ± 0.7)% for *w* = 2.0 *μ*m, (33.0 ± 0.7)% for *w* = 1.0 *μ*m, and (32.8 ± 2.0)% for *w* = 0.9 *μ*m. The visibility values cannot be calculated for *w* = 2.0–2.7 *μ*m as an interference pattern was not observed. The average intensity for the measurements performed with *w* = 2.0 *μ*m was higher than that for *w* = 1.0 *μ*m or *w* = 0.9 *μ*m [(6.72 ± 0.14) × 10^−3 ^s^−1 ^*μ*m^−2^ versus (3.26 ± 0.14) × 10^−3 ^s^−1 ^*μ*m^−2^ and (2.66 ± 0.14) × 10^−3 ^s^−1 ^*μ*m^−2^, respectively]. Thus, the highest set-up sensitivity^24^ is achieved with *w* = 2.0 *μ*m, which was used for the tomographic scans presented in this communication.

**FIG. 3.**
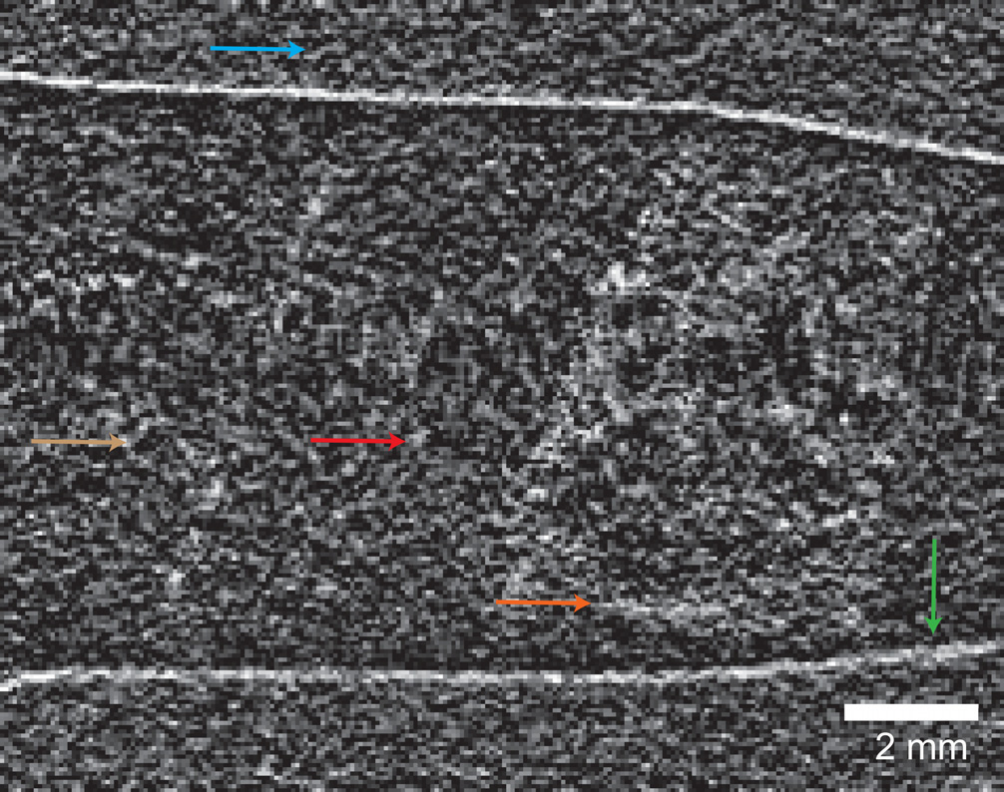
Selected cross-section of the knee sample measured using the laboratory-based double-grating interferometric set-up in the dark-field mode. Homogeneous parts, such as air (light blue), formalin (light brown), and cartilage (red), lead to negligible scattering, whereas the ones with strongly scattering structures, such as bone (orange) and container wall (green), give rise to a contrast in the dark-field mode.

XDGI is a multi-modal imaging technique, providing absorption, phase, and dark-field signals simultaneously.^30^ Homogeneous specimens, such as soft tissues, lead to negligible scattering, thus low contrast in the dark-field mode, contrary to ones with strongly scattering sub-micrometer structures such as hydroxyapatite crystallites. Therefore, in the dark-field mode, the cartilage, opposite to the bone, is hardly visible, as shown in Fig. 3.

**FIG. 4.**
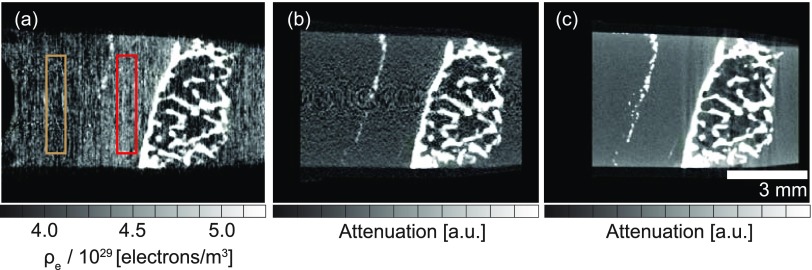
Qualitative comparison of registered cross-sections of the knee joint sample obtained in phase- and absorption-contrast modes: Laboratory-based double-grating phase contrast (a), absorption contrast with adjusted settings in order to be comparable to the grating interferometry (b) and with optimized settings for the absorption-contrast imaging (c). Color boxes (light brown color—formalin and red color—cartilage) represent the two-dimensional locations of the three-dimensional volumes of interest (VOIs) that were used for the quantitative analysis.

Figure 4 shows characteristic registered cross-sections of the knee joint sample provided by phase- and absorption-contrast modes: laboratory-based double-grating phase contrast [Fig. 4(a)], absorption contrast with adjusted settings in order to be comparable to the grating interferometry where the beam current, exposure time, sample-detector, and focus-sample distances are equivalent to the phase-contrast measurement [Fig. 4(b)] and with optimized settings for the absorption-contrast imaging with a considerably reduced scan time by increasing the tube current and decreasing the focus-detector distance [Fig. 4(c)]. The absorption-contrast measurements were performed without the double-grating interferometer. The summary of experimental parameters is listed in Table I.

**TABLE I.** Tomography settings. Phase, laboratory-based double-grating phase-contrast tomography; Absorption 1, absorption-contrast tomography with adjusted settings in order to be comparable to the grating interferometry; Absorption 2, absorption-contrast tomography with optimized settings for the absorption-contrast imaging; *U*, acceleration voltage; *I*, current; *N*, number of projections; *t*, exposure time (boldface: total exposure time for a single phase stepping image); *l*, effective pixel size; *d_p_*, sample-detector distance; and *d_o_*, focus-sample distance.

Scan	*U*	*I*	*N*	*t*	*l*	*d_p_*	*d_o_*
	(kVp)	(*μ*A)		(s)	(*μ*m)	(mm)	(mm)
Phase	42	275	600	**10.0**	23.3	460.0	140.0
Absorption 1	42	275	600	70.0	23.3	460.0	140.0
Absorption 2	42	380	600	3.0	23.3	172.5	52.5

Whereas the bony part of the sample is comparable in the phase- [Fig. 4(a)] and absorption-contrast [Figs. 4(b) and 4(c)] images, the added value of the phase-contrast mode is seen in the cartilage which is invisible in the absorption-contrast measurements. Thus, extension of the laboratory system by a phase-contrast set-up may allow visualizing soft tissues, such as cartilage, in which absorption contrast is insufficient, without losing depiction of hard tissue components. While the contrast gain for soft tissue visualization between phase- and absorption-contrast modes is the expected result,^9,31–33^ the purpose of the provided comparison is to highlight the practical contrast increase due to a grating interferometer incorporated into the commercial μCT system with a transmission source.

The results of a quantitative comparison of the phase- and absorption-contrast modes are summarized in Table II. The datasets were compared for their contrast-to-noise ratio between cartilage and formalin (CNR) and an edge-based spatial resolution (SR).^34^ Despite the inferior spatial resolution of the phase-contrast data of (73 ± 6) *μ*m, it provides enough contrast (1.094 ± 0.152) to identify the cartilage layer.

**TABLE II.** Quantitative comparison of the acquired data. Phase, laboratory-based double-grating phase contrast; Absorption 1, absorption contrast with adjusted settings in order to be comparable to the grating interferometry; Absorption 2, absorption contrast with optimized settings for the absorption-contrast imaging; CNR, contrast-to-noise ratio between cartilage and formalin; SR, edge-based spatial resolution.

Scan	CNR	SR (*μ*m)
Phase	1.094 ± 0.152	73 ± 6
Absorption 1	0.073 ± 0.007	59 ± 5
Absorption 2	0.287 ± 0.003	60 ± 5

**FIG. 5.**
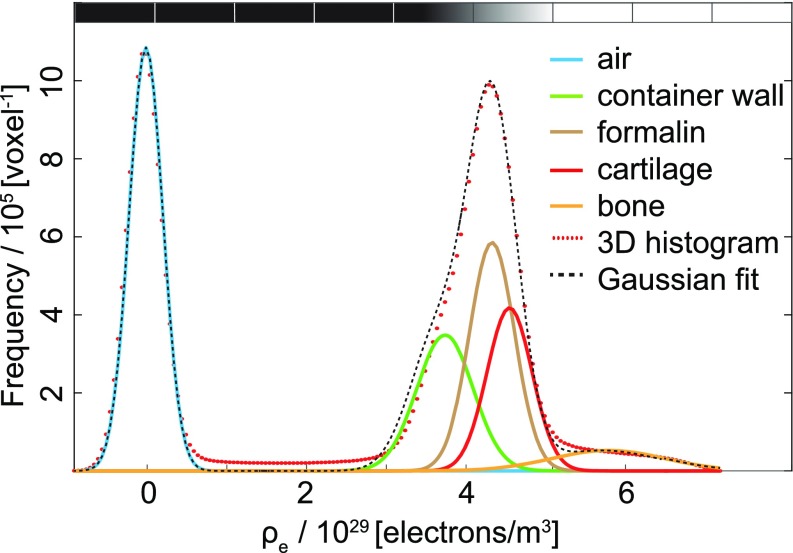
Histogram of the three-dimensional data from the knee sample measured in the phase-contrast mode with the corresponding multi-Gaussian fit. From the Gaussians related to the constituents, the electron densities of air, container, formalin, cartilage, and bone can be reasonably determined.

The advantage of XDGI is the ability to locally record the refractive index decrement, which is proportional to the electron density *ρ_e_*. The calculated value of formalin is *ρ_e,formalin_* = (4.34 ± 0.03) × 10^29^ electrons/m^3^ and of cartilage *ρ_e,cartilage_* = (4.56 ± 0.03) × 10^29^ electrons/m^3^. These values were determined as a mean intensity of a homogeneous volume of interest (VOIs) ± standard deviation, see Fig. 4(a). The histogram for the knee sample measured in the phase-contrast mode with a corresponding multi-Gaussian^35^ fit is shown in Fig. 5. From the contributions of the single constituents to the histogram, the electron densities of air, container, formalin, cartilage, and bone could be determined. The electron densities, estimated from the histogram, verify the values calculated within VOIs.

In order to calibrate the electron density measurements, several research teams performed dedicated experiments.^20,36,37^ For the present study, a cylindrical phantom consisting of the stack of polypropylene (PP), polyether ether ketone (PEEK), and polyvinyl chloride (PVC) discs with an average diameter of 6 mm and height of 1 mm each was build. The calibration procedure requires the electron densities acquired using a monochromatic X-ray beam^37^ which agrees with the calculated electron density and is not influenced by an uncertainty in the exact composition and density of phantoms. The electron densities based on the data acquired at the imaging beamline ID19 (ESRF, France),^7^ calculated as a mean intensity ± standard deviation within VOIs comprising 120 × 20 × 100 voxels within each disc, are *ρ_e,PP_* = (3.14 ± 0.01) × 10^29^ electrons/m^3^, *ρ_e,PEEK_* = (4.01 ± 0.01) × 10^29^ electrons/m^3^, and *ρ_e,PVC_* = (4.12 ± 0.01) × 10^29^ electrons/m^3^. Experimental data acquired at the imaging branchline I13-2 (Diamond Light Source, UK)^6^ verified the results. Electron densities, calculated within VOIs = 120 × 20 × 100 voxels within each disc acquired using the laboratory set-up, are *ρ_e,PP_* = (3.86 ± 0.05) × 10^29^ electrons/m^3^, *ρ_e,PEEK_* = (4.87 ± 0.07) × 10^29^ electrons/m^3^, and *ρ_e,PVC_* = (4.99 ± 0.06) × 10^29^ electrons/m^3^. In our case, the measured electron densities were overestimated with a mean offset of (0.86 ± 0.08) × 10^29^ electrons/m^3^. After subtracting the calculated offset, the local electron densities determined from the laboratory data match the expected ones with the maximal deviation of 3%.

## III. CONCLUSION AND OUTLOOK

Our results suggest that the extension of a commercially available μCT system via a grating interferometer offers potential to fill the gap between laboratory-based absorption-contrast μCT and phase-contrast μCT using synchrotron radiation or conventional X-ray sources in visualizing soft tissues. We demonstrated an improvement in the contrast resolution when compared to an absorption-contrast mode and quantitative accuracy of XDGI, showcasing the complementarity of the set-up.

In the future, the most important further step for biomedical applications would be the increase in the sensitivity of the grating interferometer. For this purpose, an asymmetric set-up working at a higher fractional Talbot order with a decreased period of the analyzer grating will be realized. As it was previously shown, synchrotron radiation-based grating interferometry enables label-free imaging of a human knee down to the cellular level.^6^ It is expected that further sensitivity increase of the laboratory set-up can enable visualizing individual chondrocytes and potentially their automatic counting, which in turn will lead to clear detection of individual cartilage layers.

## IV. METHODS

### A. Specimen preparation

A human knee joint sample obtained *post-mortem* from an 87-year-old female body donated to the Institute of Anatomy, University of Basel, Switzerland, was investigated. All donors of the program contributed their body to education and research purposes. All procedures were conducted in accordance with the Declaration of Helsinki. Experts extracted the knee piece 5.4 mm in diameter and 4.0 mm in height from the surface of the tibia around the contact area of the femoro-tibial joint, where a thick cartilage layer (∼2 mm) was preserved, subsequently fixed in 4% buffered formalin, and kept in a formalin-filled Eppendorf container.

In order to verify the calibration procedure feasibility for estimating the electron densities, the cylindrical phantom consisting of the stack of polypropylene (PP), polyether ether ketone (PEEK), and polyvinyl chloride (PVC) discs with an average diameter of 6 mm and height of 1 mm was build.

### B. Micro computed tomography

The tomography measurements were carried out in an advanced absorption-contrast μCT system nanotom^®^ m equipped with a 180 kV/15 W nanofocus^®^ transmission tube with an adjustable focal spot diameter *w* (0.9–2.7 *μ*m) and operating with a tungsten target.^38^ The system has a temperature stabilized digital GE DXR 500L detector with a pixel size of 100 *μ*m (3072 × 2400 pixels) based on endurance™ scintillator technology.^39^ Experimental parameters are listed in Table I. The scanning parameters for Absorption 1 were adjusted in order to be comparable to the grating interferometry although they were not optimal for the absorption-contrast imaging. The scanning parameters for Absorption 2 were optimized for the absorption-contrast imaging. All tomography scans were performed with a focal spot diameter *w* = 2.0 *μ*m. For the absorption-contrast measurements, data processing and reconstruction were done automatically, using datos|x 2.0 software (phoenix|x-ray, GE Sensing & Inspection Technologies GmbH, Wunstorf, Germany), which implements cone-beam reconstruction based on the Feldkamp algorithm.^40^

A double-grating symmetric interferometric set-up was incorporated into the μCT system nanotom^®^ m. The measurements were performed using a phase grating G1 with a periodicity of 7 *μ*m and a gold structure height of 6 *μ*m, in order to induce a phase shift of *π* for the photon energy of 30 keV, corresponding to an estimated acceleration voltage of 42 kVp.^28^ The gold lines of an analyzer grating G2 had a structure height of 85 *μ*m with a periodicity of 7 *μ*m. The inter-grating distance corresponds to the first fractional Talbot order, where the phase grating G1 is positioned at a distance of *d*_1_ = 29.6 cm downstream from the source and the analyzer grating just in front of the detector *d*_2_ = 29.6 cm. A phase-stepping technique, which allows the extraction of phase, absorption, and dark-field signals,^41^ where G2 was scanned over two periods of the interference pattern in seven phase steps, was used. For the tomography, 600 equi-angular projections over 360° with the total exposure time per single phase stepping image of 10 s (2 × 5 s) were acquired. Reference (without a sample) and dark (without X-ray beam) images were taken every 50 angular positions. Additional experimental parameters are listed in Table I. The phase recovery and parallel-beam reconstruction using the filtered back-projection algorithm^42,43^ with a modified filter kernel (Hilbert transform) of the data were carried out using MATLAB^®^ (The Mathworks Inc., Nattick, USA). The raw data were median-filtered for noise reduction (kernel size 3 × 3). To correct for the cone-beam geometry, phase projections were renormalized^44^ by a factor of $\frac{d_{1}}{d_{o}}=2.11$, where *d_o_* denotes the focus sample distance. The measurement of the phantom was performed with an effective pixel size of 23.1 *μ*m. After reconstruction, the acquired data were median filtered (kernel size 3 × 3) for noise reduction.

We have compared a parallel-beam reconstruction using the filtered back-projection algorithm with Hilbert filter implemented in MATLAB^®^ and an optimized cone-beam reconstruction based on the Feldkamp algorithm implemented in datos|x 2.0. For the quantitative comparison of the datasets, translation registration using the library provided by Insight Segmentation and Registration Toolkit (ITK)^45^ was used. We have verified that the reconstruction using the assumption of the parallel- or cone-beam has a negligible effect on the final result. Both reconstructions yielded almost identical results in terms of the density and spatial resolutions.

The synchrotron radiation based XDGI measurements were carried out at the Diamond-Manchester Imaging Branchline I13-2 (Diamond Light Source, UK)^6^ at a photon energy of 19 keV, an inter-grating distance of *d*_2_ = 480 mm (eleventh Talbot order), an exposure time of 5 s per phase-stepping image for 900 equi-angular positions, and an effective pixel size of 2.3 *μ*m and at the imaging beamline ID19 (ESRF, France)^7^ at a photon energy of 52 keV, an inter-grating distance of *d*_2_ = 360 mm (first Talbot order), an exposure time of 0.5 s per phase-stepping image for 200 equi-angular positions, and an effective pixel size of 5.1 *μ*m. Additional imaging and reconstruction parameters are identical to the ones used by Schulz *et al.*^6,7^

### C. Quantitative evaluation

The quantitative comparison was carried out in MATLAB^®^ and was based on the determination of a contrast-to-noise ratio between cartilage and formalin (CNR) and an edge-related spatial resolution (SR). For the quantitative comparison of the datasets, the rigid registration with nearest-neighbor interpolation using the library provided by ITK was used. The CNR was defined as $|I_{\text{cartilage}}-I_{\text{formalin}}|/\sigma_{\text{formalin}}$, where *I* denotes the mean intensity of a homogeneous volume of interest (VOI) and *σ* is the standard deviation. The VOI, which corresponds to 124 × 20 × 24 voxels, was selected within the cartilage and the formalin from the same virtual location within each registered dataset, see Fig. 4(a). The intensity distribution within VOI was fitted with Gaussian^35^ using Curve Fitting App^®^ to extract *I* and *σ*. SR was defined as the intersection of the normalized modulation transfer function (nMTF) with its 10% value, and for its estimation, a region at the bone-cartilage interface was chosen. In order to reduce noise effects, the profile line was smoothed using a moving average filter with a span of five.

The histogram of the three-dimensional data from the knee sample measured in the phase-contrast mode was fitted a multi-Gaussian function by means of Curve Fitting App^®^. From the Gaussians related to the constituents, the electron densities of air, container, formalin, cartilage, and bone can be reasonably determined. The electron densities for air, cartilage, and formalin, as previously estimated within VOIs, were used as the starting points for the locations of Gaussian peaks.
